# Abnormal NFAT5 Physiology in Duchenne Muscular Dystrophy Fibroblasts as a Putative Explanation for the Permanent Fibrosis Formation in Duchenne Muscular Dystrophy

**DOI:** 10.3390/ijms21217888

**Published:** 2020-10-24

**Authors:** Sandrine Herbelet, Boel De Paepe, Jan L. De Bleecker

**Affiliations:** 1 Department of Neurology, Ghent University and Ghent University Hospital, C. Heymanslaan 10, 9000 Ghent, Belgium; Boel.DePaepe@UGent.be (B.D.P.); Jan.DeBleecker@UGent.be (J.L.D.B.); 2 Neuromuscular Reference Center, Ghent University Hospital, C. Heymanslaan 10, 9000 Ghent, Belgium

**Keywords:** NFAT5, Duchenne muscular dystrophy, fibroblasts, hyperosmolar or pro-inflammatory cell stress

## Abstract

Duchenne muscular dystrophy (DMD) is characterized by chronic inflammation and fibrotic tissue production by fibroblasts. The promyogenic factor nuclear factor of activated T-cells 5 (NFAT5) is virtually present in all cells, responding to hyperosmolar or pro-inflammatory stress. In embryogenic fibroblasts, absence of NFAT5 results in cell cycle arrest. Here, unaffected skeletal muscle fibroblasts from one healthy donor showed NFAT5 nuclear translocation upon hyperosmolar stress and normal cell viability. Absence of NFAT5 translocation under pro-inflammatory conditions resulted in decreased cell growth (Incucyte ZOOM). In DMD skeletal muscle fibroblasts from one DMD patient, NFAT5 was merely located in the nucleus. Exposure to hyperosmolar conditions or pro-inflammatory cytokines IFN-γ, IL-1β and TNF-α had no influence on NFAT5 physiology (immunofluorescence, western blotting, RT-qPCR). Hyperosmolarity resulted in decreased cell viability and pro-inflammatory stress in unaltered cell growth. These findings suggest that NFAT5 is vital to DMD fibroblast survival. Exposure to pro-inflammatory or hyperosmolar stress in DMD fibroblasts results in an unexpected NFAT5 response, where fibroblasts are not triggered by inflammatory cytokines and do not withstand hyperosmolarity. Chronic inflammation could be viewed as a non-restrictive factor in the formation of fibrosis in DMD. Abnormal NFAT5 physiology could provide a molecular explanation for permanent fibrotic matrix production by DMD fibroblasts.

## 1. Introduction

Duchenne muscular dystrophy (DMD) is an inherited, X-linked, severe muscle degenerating disorder caused by mutations in the dystrophin gene as initially described in 1987 by Hoffman et al. [1] and Koenig et al. [2]. This leads to dysfunctional dystrophin-associated protein complex (DAPC), contraction-induced damage and leaky channels in myocytes [3,4]. Sarcolemmal changes allow Ca²^+^ and Na^+^ entry, and consequently, increased intracellular ionic concentration [5,6]. Perturbed cellular pathways due to DAPC instability further hamper myocyte homeostasis [7]. DMD is characterized by cycles of myocyte degeneration and regeneration, where muscular tissue is ultimately replaced by fibrotic tissue. The latter is formed by fibroblasts, which initially play a scaffolding role, along which skeletal muscle stem cells, called myoblasts, regenerate the injured tissue. This process is a tightly controlled process, where fibroblasts disappear when their scaffolding role is no longer needed. However, in chronic inflammatory conditions, fibroblasts will remain and further produce fibrotic tissue, i.e., stiff scar tissue that has a negative influence on patients’ mobility [8,9,10].

The transcription factor of activated T cells 5 (NFAT5) is important for cell survival in mouse embryogenic fibroblasts. Absence of NFAT5 in this cell type leads to loss of function [11]. In cells exposed to hyperosmolar changes such as kidney cells, the major task of NFAT5 consists of maintaining cellular homeostasis by translocation of NFAT5 [12,13,14] and activation of protective genes upon exposure to a hyperosmolar extracellular environment [15]. Production of organic osmolytes will restore cellular homeostasis. This consists of attracting water back into the cell, which was lost by osmosis to the extracellular environment [16]. The NFAT5 pathway can be activated by two distinct stimuli: osmotic shock or receptor activation. The cell can discriminate by which stimulatory pathway NFAT5 has been activated and upregulated, yielding a different gene activation program. Besides organic osmolyte induction, NFAT5 can activate tumor necrosis factor (TNF) and heat shock protein 70 (HSP70) transcription [17,18].

In a previous study, the effect of hyperosmotic conditions on aldose reductase, regulated by NFAT5 in response to cellular hyperosmolarity, was investigated in DMD myoblasts. The expression levels of aldose reductase, which is a source of the cytoprotective osmolyte sorbitol, was observed to be no different between untreated cells and cells exposed to hyperosmolar stress, probably due to permanent intracellular increased ionic stress and subsequent permanent activation of NFAT5 in DMD myoblasts [19]. With NFAT5 playing a role also in fibroblast function, DMD being characterized by chronic inflammation and DMD patients suffering permanent production of fibrotic tissue in skeletal muscles, we studied the localization and expression of NFAT5 in healthy and DMD skeletal fibroblasts of one healthy individual and one affected by DMD, after exposure to hyperosmolar or pro-inflammatory stress.

## 2. Results

### 2.1. NFAT5 Translocates to the Nucleus in Unaffected Fibroblasts Exposed to Hyperosmolar Stress

**Figure 1:**
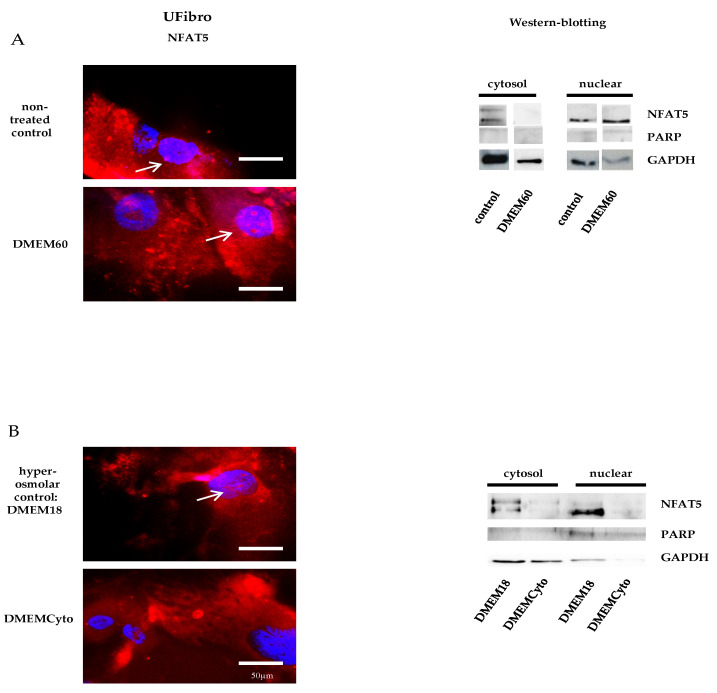
Nuclear factor of activated T-cells 5 (NFAT5) physiology in unaffected fibroblasts exposed to hyperosmolar or pro-inflammatory stress. In red, localization of NFAT5 is visualized. Myonuclei are stained with DAPI (blue) (*n* = 3). NFAT5 translocates to the nucleus in unaffected fibroblasts exposed to hyperosmolar stress, as indicated by the white arrows (DMEM60) (**A**) and remains in the cytoplasm under pro-inflammatory stimulation (DMEMCyto) (**B**). A hyperosmolar control (DMEM18) was used in B to study the effect of DMEMCyto only.

**Figure 2:**
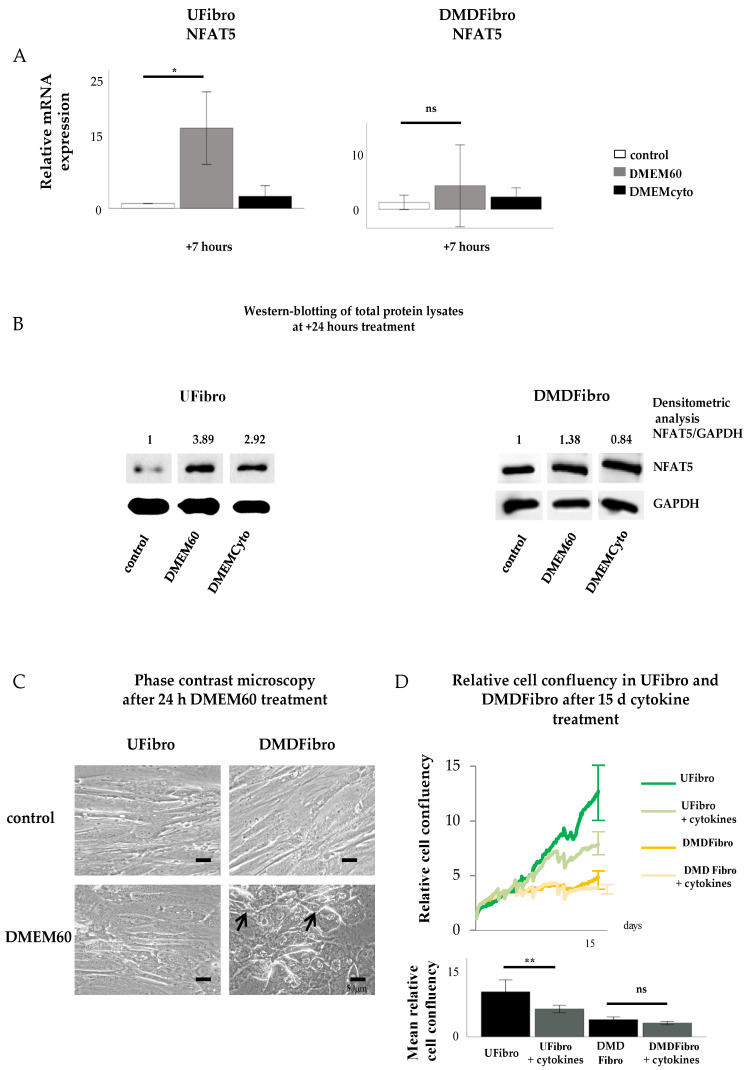
NFAT5 mRNA, protein expression and cell growth in unaffected and DMD fibroblasts exposed to hyperosmolar or pro-inflammatory stress. Normal NFAT5 physiology in unaffected fibroblasts induce delayed cell growth when exposed to pro-inflammatory stress (DMEMCyto) (*p* < 0.01) (**A**–**D**), whereas abnormal NFAT5 physiology in DMD fibroblasts leads to cell detachment in hyperosmolar medium (DMEM60) and unaffected cell growth under pro-inflammatory setting (DMEMCyto) (**A**–**D**). * *p* < 0.05, ** *p* < 0.01, ns indicates no significance.

Unaffected fibroblasts (UFibro) showed a normal NFAT5 physiological reaction towards both pro-inflammatory cytokines and hyperosmolar stress. At rest, NFAT5 was mainly localized in the cytoplasm and could be discretely observed in the nuclei of UFibro (Figure 1A). Exposure to hyperosmolar NaCl (DMEM60) induced translocation of NFAT5 to the nucleus, followed by increased NFAT5 mRNA expression in the first 7 h post stimulation and increased NFAT5 protein expression at + 24 h (Figure 1A and Figure 2A,B). Nuclear translocation was clearly visible by immunofluorescence (IF) (Figure 1A, left side, white arrow) and by fractionated western blotting (WB) where NFAT5 decreased in the cytoplasmic fraction and increased in the nuclear compartment (Figure 1A, right side). After exposure to pro-inflammatory cytokines IL-1β, TNF-α and IFN-γ (DMEMCyto), NFAT5 remained in the cytoplasm (Figure 1B) and NFAT5 mRNA and protein expression were not increased (Figure 2A,B).

### 2.2. NFAT5 was Largely Nuclear in Untreated DMD Fibroblasts and Did Not Respond Further to Hyperosmolar Stress

**Figure 3:**
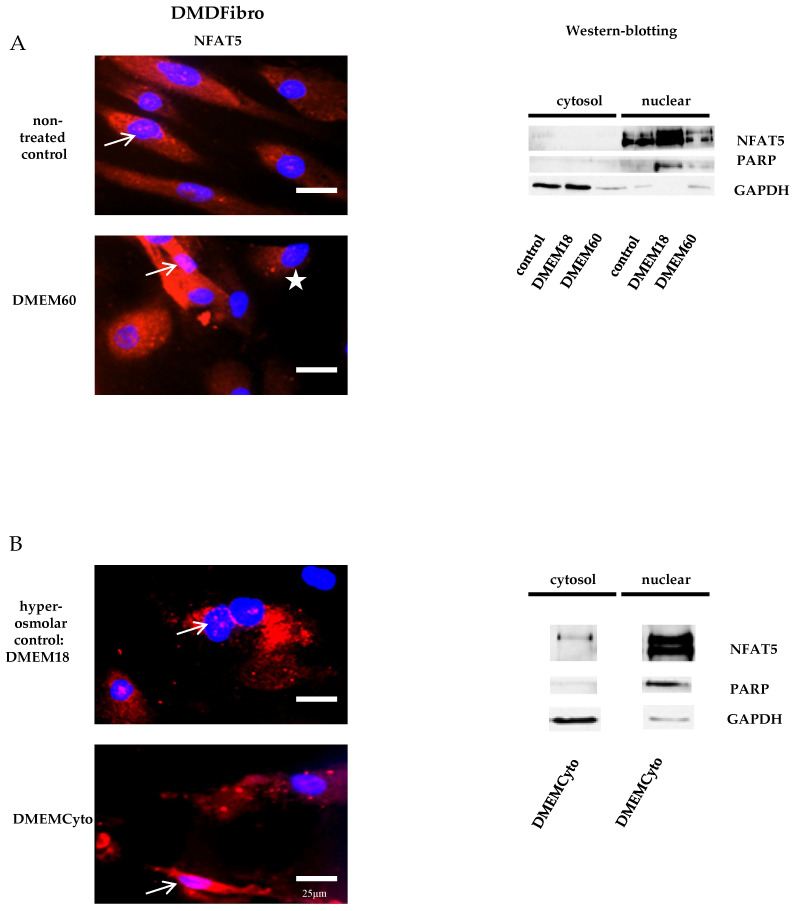
NFAT5 physiology in DMD fibroblasts exposed to hyperosmolar or pro-inflammtory stress. In red, localization of NFAT5 is visualized. Myonuclei are stained with DAPI (blue) (*n* = 3). NFAT5 was largely nuclear in untreated DMD fibroblasts, as indicated by the white arrows (**A**,**B**) and did not respond further to hyperosmolar or pro-inflammatory stress (**A**,**B**). In some DMDFibro exposed to hyperosmolar NaCl, NFAT5 was not present in the cytoplasm and in the nucleus (with star) (**A**).

Fibroblasts donated by a DMD patient (DMDFibro) displayed unexpected NFAT5 behavior after exposure to pro-inflammatory cytokines and hyperosmolar stress. In unstimulated DMDFibro, NFAT5 was faintly present in the cytoplasm and more clearly present in nuclei compared to UFibro as indicated by the white arrow in IF and strong presence in the nuclear compartment of fractionated WB (Figure 3A, left and right side). Exposure to DMEM60 did not yield a uniform NFAT5 expression pattern by IF (Figure 3A, left side). Some cells showed a very clear nuclear NFAT5 staining (white arrow), while other cells had almost no NFAT5 nuclear staining (white star) (Figure 3A). In fractionated WB, almost all NFAT5 was located in the nuclear compartment, both at rest and after hyperosmolar NaCl stress (Figure 3A, right side). Despite this observation, NFAT5 mRNA and protein expression where not significantly increased (Figure 2A,B). The repercussion of these effects was visible in the cell morphology of DMDFibro, as observed under the phase contrast microscope at + 24 h post stimulation with DMEM60 (Figure 2C). Cells started to show signs of cell detachment (black arrows). Exposure to DMEMCyto revealed most of NFAT5 being present in the nuclear compartments, both by IF and fractionated WB (Figure 3B, left side, white arrow and right side). However, NFAT5 mRNA and protein expression remained unchanged (Figure 2A,B).

### 2.3. Pro-Inflammatory Cytokines Decreased Cell Growth of Control Fibroblasts, but Not of DMD Fibroblasts

Cytokine-treated UFibro had unchanged morphology, as observed under the phase contrast microscope at + 24 h stimulation with DMEM60 (Figure 2C). Addition of DMEMCyto for 15 d revealed a significant reduction in cell growth as monitored by Incucyte ZOOM and expressed in relative cell confluency with *p* < 0.006 (Figure 2D). In contrast, DMDFibro were not affected by DMEMCyto stress for 15 d as monitored by Incucyte ZOOM (Figure 2C). No statistical difference could be observed between the DMDFibro control group and the DMDFibro group exposed to DMEMCyto (Figure 2C).

In conclusion, unaffected skeletal muscle fibroblasts have the possibility to react in a normal physiological manner to hyperosmotic or pro-inflammatory stress by translocating NFAT5 to their nucleus under hyperosmolar conditions and not proceeding to this step in a pro-inflammatory setting. At cellular growth level, these cells will be able to withstand hyperosmolarity but not pro-inflammatory environments. On the contrary, skeletal muscle fibroblasts, found in DMD tissue, have a perturbed NFAT5 response. During both hyperosmolar and pro-inflammatory stress, NFAT5 resides in the nucleus and does not seem able to react to this kind of stimulus, resulting in cell death under strong hyperosmolar conditions and unaltered cell growth in a pro-inflammatory setting. The latter may explain the presence of continuous fibrosis formation in DMD where permanent inflammation is prevalent.

## 3. Discussion

DMD is characterized by aberrant fibrosis deposition resulting in hampered muscle functioning [20]. In rheumatoid arthritis (RA), a disease characterized by high levels of pro-inflammatory cytokines, NFAT5 is translocated to the nucleus and highly expressed in fibroblast-like synoviocytes (FLS) after exposure to pro-inflammatory cytokines, including TNF-α and IL-1β. In FLS, NFAT5 is a critical factor in the regulation of their proliferation in RA. FLS become resistant to apoptosis during the disease and actively participate by producing pro-inflammatory cytokines [21]. In this study we observed that pro-inflammatory cytokines IFN-γ, IL-1β and TNF-α did not induce NFAT5 nuclear translocation and activation in DMD fibroblasts. Instead, NFAT5 appeared to be located at rest in the DMD fibroblast nucleus and seemed to not react to pro-inflammatory stimuli. Cell growth over 15 d was unchanged compared to control fibroblasts exposed to the same pro-inflammatory conditions. In DMD fibroblasts, NFAT5 seems unresponsive to hyperosmolar stimuli as well. This was reflected in decreased cell viability after exposure to DMEM60, showing the importance of NFAT5 in fibroblast survival. This could explain the increased Na^+^ concentration in mdx muscle tissue [22], as NFAT5 is not able to regulate cell volume homeostasis, and subsequently, Na^+^ concentrations in the cell. In healthy cells, NFAT5 is the master regulator of osmolarity, by sensing hyperosmolar environments and protecting cells from it, by activating genes coding for organic osmolytes. These osmolytes harbor the possibility to restore cell homeostasis, by draining water back to the cell which has diffused to the extracellular environment under the passive force of osmosis [12]. Absence of reaction of NFAT5 to both hyperosmolar and pro-inflammatory stress in DMD fibroblasts could possibly be explained by a changed conformation of the nuclear localization signal (NLS) and/or the nuclear export signal (NES). Indeed, NFAT5 has a bipartite NLS in its N-terminal region, which is unveiled upon phosphorylation, allowing NFAT5 translocation into the nucleus. NFAT5 possesses several amino acids which can be phosphorylated, such as serine 155 and 158 (S155 and S158), tyrosine 143 (Y143) and threonine 135 (T135) [23,24].

In muscle regeneration, the timely coordinated ballet of fibroblasts and myoblasts is essential to proper muscle reconstruction [25]. The switch from pro-inflammatory macrophages, which clean up the debris, to the anti-inflammatory phenotype, which help rebuild the tissue, is essential. Fibroblasts form a scaffold along which myoblasts can grow, disappearing at the right moment to avoid fibrosis. Pro-inflammatory macrophages produce pro-inflammatory cytokines such as TNF-α and IL-1β [26]. These are the cytokines used in our model. Interestingly, dermal fibroblasts exposed to TNF-α for up to 20 days undergo premature senescence through activation of p38-MAPK and reactive oxygen species (ROS). Inhibition of the p38-MAPK pathway rescue dermal fibroblasts from senescence [27]. NFAT5 and p38-MAPK are closely linked to each other [28]. Therefore, it could be conceivable that in skeletal muscle fibroblasts, this phenomenon is also present. This could explain the decrease in cell growth observed in skeletal muscle-derived fibroblasts exposed to TNF-α, IL-1β and IFN-γ in this study. At day 15, a statistically significant difference was observed (*p* < 0.006). The following mechanism could explain this phenomenon: activation of p38-MAPK could induce its interaction with NFAT5, thereby avoiding NFAT5 nuclear translocation. With presence of NFAT5 in the nucleus being essential to fibroblast cell growth, this could explain senescence. This mechanism could be a safeguard as to avoid fibrosis during tissue repair. In this study, NFAT5 remains in the nucleus of fibroblasts issuing from DMD. This could imply that p38-MAPK and NFAT5 are not able to communicate with each other and that senescence cannot occur. This could explain the unaltered cell growth observed in this study in DMD fibroblasts. Indeed, no significant difference was observed between DMD fibroblast with or without exposure to pro-inflammatory cytokines.

TNF-α was used at a dosage of 10 ng/mL in this study, whereas dosages of physiological TNF-α during myogenesis are estimated around several pictograms per mL. TNF-α dosages of 10 to 20 ng/mL, as seen in chronic inflammation, have been shown to inhibit myogenesis [29]. Results from this study suggest a role for NFAT5 in regulating cell death in skeletal muscle fibroblasts. TNF-α might play an important role herein by modulating p38-MAPK and maintaining NFAT5 localized in the cytoplasm at high dosages/during chronic inflammation. In DMD fibroblasts, where NFAT5 seems mainly located in the nucleus, this phenomenon could not occur by the absence of communication between p38-MAPK and NFAT5, thereby bypassing a possible natural anti-fibrosis mechanism, and partially explaining the permanent fibrotic tissue production and deposition seen in DMD, which is harmful to patients.

## 4. Materials and Methods

### 4.1. Cell Line Identification, In Vitro Cell Culture and Testing Conditions

Healthy and DMD fibroblasts were obtained from the Myobank Banque d’ADN (Paris, France) (Table S1) and identification was performed by submitting cell pellets to Eurofins Genomics (Eurofins Scientific Group, Luxemburg, Luxemburg) for cell line genotyping following ANSI/ATCC standard ASN-0002. Cell line identification was performed using 16 DNA markers, the Applied Biosystems^TM^ AmpFLSTR^TM^ Identifiler^TM^ Plus PCR Amplification Kit system (ThermoFisher Scientific, Waltham, MA, USA) and an ABI capillary sequencer with STR markers for CSF1PO, D2S1338, D3S1358, D5S818, D7S820, D8S1179, D13S317, D16S539, D18S51, D19S433, D21S11, FGA, TH01, TPOX and vWA and the gender marker Amelogenin. Certificates are included in Supplementary Materials (Figure S1).

After local Ethic Committees approval, Myobank’s healthy skeletal muscle fibroblasts obtained from a healthy donor (“UFibro”) and DMD skeletal muscle fibroblasts donated by a DMD patient (“DMDFibro”) were grown each in three different passages (*n* = 3) (Table S1). These fibroblasts originated from skeletal muscle biopsies. Nutrients vital to cell growth were included in DMEM. This medium was supplemented with 10% Fetal Calf Serum FCS (Cambrex, Bioscience, Walkersville, MD, USA), penicillin (50 IU/mL) + streptomycin (50 mg/mL) (Gibco, Invitrogen, Carlsbad, CA, USA), glucose and 1% L-glutamine (Life technologies, Carlsbad, CA, USA). All cell cultures were monthly checked for Mycoplasma with the MycoAlert Plus Kit, with all tests negative on contamination (Lonza, Basel, Switzerland). Cells were grown to confluency in 75-cm^2^ flasks for WB and RT-qPCR, one flask per condition. For further testing and evaluation under the phase contrast (Leica, DMI3000 B, Wetzlar, Germany) and confocal microscope, both UFibro and DMDFibro were cultured in 8-well chamber slides (LabTek II, Nunc, Penfield, Hudson, MA, USA).

At confluence, UFibro and DMDFibro were exposed to hyperosmolar or pro-inflammatory conditions. Briefly, fibroblasts were exposed to a mixture of pro-inflammatory cytokines IFN-γ with IL-1β and TNF-α diluted in DMEM (“DMEMCyto”) to respective concentrations of 300 U/mL, 20 ng/mL and 30ng/mL for 7 h and 24 h (R&D Systems, Minneapolis, MN, USA) [30]. This corresponds to the inflammatory conditions seen in DMD. Non-supplemented DMEM had an osmolarity of 110 mM (280 mOsm/L). Cytokine supplementation slightly increased DMEM’s osmolarity to 128 mM (333 mOsm/L). Therefore, to study the effect of cytokines, the control DMEM medium was supplemented with 18 mM NaCl (“DMEM18”) [31].

Hyperosmolar conditions were obtained by adding 3.50 mg/mL NaCl to DMEM, obtaining a 60 mM increase of the solution (“DMEM60”), resulting in a final osmolarity of 170 mM (443 mOsm/L). Addition of NaCl was performed as to mimic Na^+^ concentration measured in DMD patients’ fibers [32]. DMEM contains 110 mM NaCl, and Weber et al. described in DMD patients a normalized muscular isotope ^23^Na increase with 38.4 ± 6.8 mmol/L compared with 25.4 ± 2.1 mmol/L in healthy volunteers (*p* < 0.001). A maximum increase of 21.9 mM in Na^+^ was, thus, measured. A similar increase was measured in mdx mice, in the murine DMD model by Dunn et al. [22]. Therefore, addition of 18 mM NaCl to DMEM was applied. Addition of 60 mM NaCl was chosen based on the Na^+^ concentration in blood (135–145 mM) in combination with increased salt intake; in 2006, Yoshida et al. described the benefit of such a diet in *mdx* mice. These mice expressed less muscle necrosis than *mdx* mice that did not receive this increase in salt uptake [33]. Both cell lines were exposed to hyperosmolar conditions for 7 h or 24 h. All osmolarity measurements were performed using an osmometer (Osmometer Automatic, Knauer, RS45105-0, Berlin, Germany).

### 4.2. Life Imaging by Incucyte ZOOM and Phase Contrast Microscopy

Fibroblasts were seeded at high densities: UFibro and DMDFibro (*n* = 3) were seeded in a 24-NUNC well plate (Nalge Nunc International, Rochester, NY, USA) (4000 cells/well, 1 mL/well and 1 well = 2 cm^2^) and allowed to adhere overnight at 5% CO_2_ and 37°C. The plate was placed following the manufacturer’s guidelines in the IncuCyte ZOOM (Essen Bioscience, Hertfordshire, UK), allowing real-time monitoring of cell confluence over time. Cells were exposed to the cytokines TNF-α (30 ng/mL), IFN-γ (300 U/mL) and IL-1β (20 ng/mL). Relative cell confluence was obtained by normalizing values to 1, thereby allowing to compare cell lines.

Survival over several days under hyperosmolar conditions appear difficult for fibroblasts, especially when issuing from DMD muscle. Therefore, morphology was assessed by using a phase contrast microscope at time point + 24 h.

### 4.3. RT-qPCR

Total lysis of 75-cm^2^ flasks was performed following a well-described methodology and with a minimum purity of 1.9/2 measured by the A260/A280 ratio on BioDrop™ Touch Duo PC (Harvard Bioscience Inc., Holliston, MA, USA) [34]. Minimum information for publication of quantitative real-time PCR experiments (MIQE) guidelines [35] were applied and results were obtained by calculation with qBase+ Software version 2.6 (www.qbaseplus.com) (Biogazelle, Zwijnaarde, Belgium) [36]. geNorm was used to determine the three most stable reference genes from a set of tested candidate reference genes (Table S2).

### 4.4. Quantitative and Fractionated Western Blotting (WB)

After transfer to nitrocellulose membranes by electroblotting, proteins from total protein extracts were incubated with primary antibodies (Table S3). Anti-GAPDH (Sigma-Aldrich, St. Louis, MO, USA) was used to correct for protein concentration between samples. Immunoreaction was detected using chemiluminescence (WesternBright™ Sirius, Advansta, Menlo Park, CA, USA) and Proxima 2650 (Isogen Life Science, De Meern, The Netherlands). NFAT5 antibody selectivity for WB was assessed using siRNA NFAT5 (h): sc-43968 (Santa Cruz Biotechnology, Dallas, TX, USA) in DMDFibro, as described earlier and included in Supplementary Materials (Figure S2). Fractionated WB were performed, as thoroughly described in an earlier work [31].

### 4.5. Immunocytochemistry (ICC) and Confocal Microscopy (CM)

Staining and specificity tests were performed as described previously [31]. Primary antibody was used for one hour followed by a secondary antibody labelled with AlexaFluor-488 (green) or AlexaFluor-555 (red) (Invitrogen, Waltham, MA, USA) also for one hour. Antibody and concentration used are listed in Supplementary Table S4.

Confocal images were captured with a Zeiss LSM880 confocal microscope (Zeiss, Jena, Germany). Images were taken using a 40×Plan-Apochromat/1.3 oil objective, with the pinhole set at 1 Airy Unit.

### 4.6. Statistical Analysis

For all results, mean and standard deviation was calculated for each passage at each time point. This was performed for all conditions (control, hyperosmolar or pro-inflammatory conditions) using SPSS 26.0 (IBM, Armonk, NY, USA). Means and standard deviations from three passages (biological replicates) resulted in one mean and one standard deviation by using a one-way ANOVA with Tukey’s multiple comparison test. Following significant values were considered: * = *p* < 0.05, ** = *p* < 0.01, *** = *p* < 0.005.

## Supplementary Materials

Supplementary materials can be found at https://www.mdpi.com/1422-0067/21/21/7888/s1. Figure S1: Certificates. Figure S2: Testing of NFAT5 Mouse specificity using siRNA. Table S1: Cell culture data. Table S2: Genes used in RT-qPCR. Table S3: Primary antibodies used in Western-blotting. Table S4: Primary antibodies used in immunecytohistochemistry.

## Abbreviations

CM	confocal microscopy
DAPC	dystrophin-associated protein complex
DMD	Duchenne muscular dystrophy
DMDFibro	Duchenne muscular dystrophy fibroblasts
DMED18	DMEM supplemented with 18 mM NaCl
DMEM60	DMEM supplemented with 60 mM NaCl
DMEMCyto	DMEM supplemented with IL-1β + TNF-α + IFN-γ
ECM	extracellular matrix
FCS	Fetal Calf Serum
FLS	fibroblast-like synoviocytes
GAPDH	glyceraldehyde-3-phosphate dehydrogenase
HSP70	heat shock protein 70
ICC	immunocytochemistry
IF	immunofluorescence
MIQE	minimum information for publication of quantitative real-time PCR experiments
NES	nuclear export signal
NLS	nuclear localization signal
N	number of passages
NFAT5	nuclear factor of activated T-cells 5
RA	rheumatoid arthritis
RT-qPCR	real-time quantitative PCR
siRNA	silencing RNA
TNF	tumor necrosis factor
UFibro	unaffected fibroblasts (from skeletal muscle)
WB	western blotting
