# Supplementary material for: Abnormal NFAT5 Physiology in Duchenne Muscular Dystrophy Fibroblasts as a Putative Explanation for the Permanent Fibrosis Formation in Duchenne Muscular Dystrophy
Source: Int J Mol Sci. 2020 Oct 24;21(21):7888. doi: 10.3390/ijms21217888 (PMC7660673; doi:10.3390/ijms21217888)

APG20_PR0154_CLA_Herbelet_WDH

Sample File	Sample Name	Panel	SQI	OS	SQ
F01_20_ZE_000261v5_APG20_PR0154.fsa	20_ZE_000261v5	Identifiler_Plus_Panels_v1			

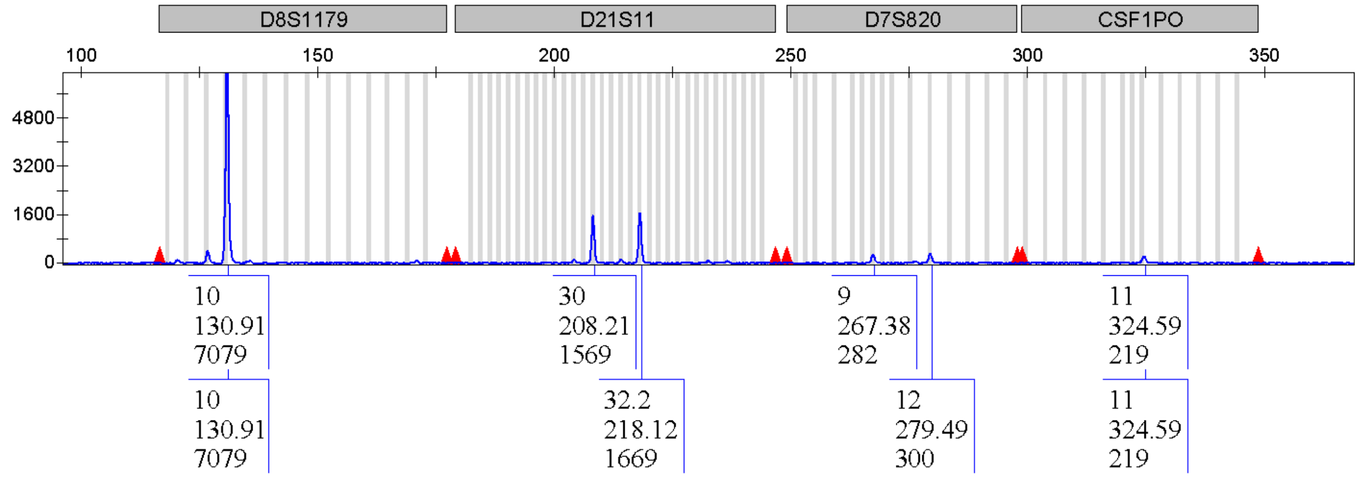

F01_20_ZE_000261v5_APG20_PR0154.fsa	20_ZE_000261v5	Identifiler_Plus_Panels_v1			

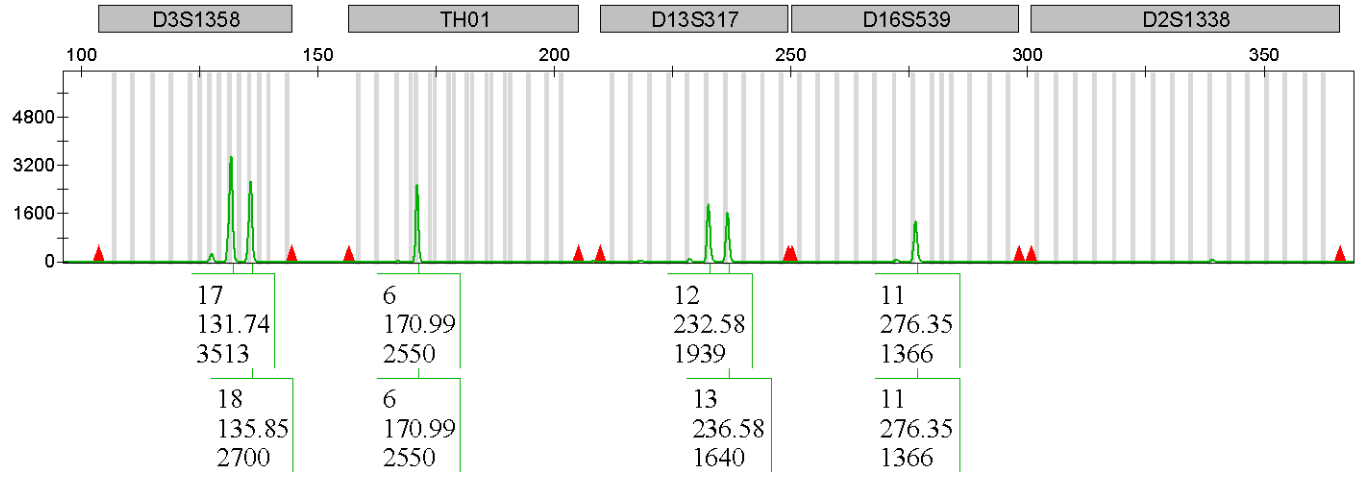

F01_20_ZE_000261v5_APG20_PR0154.fsa	20_ZE_000261v5	Identifiler_Plus_Panels_v1			

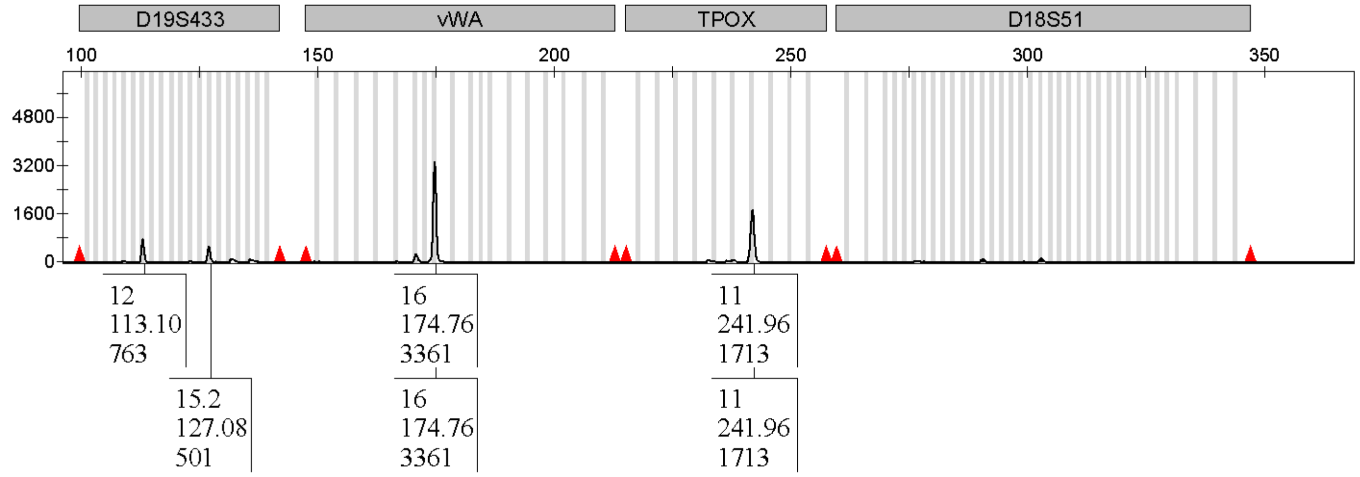

Sample File	Sample Name	Panel	SQI	OS	SQ
F01_20_ZE_000261v5_APG20_PR0154.fsa	20_ZE_000261v5	Identifiler_Plus_Panels_v1			

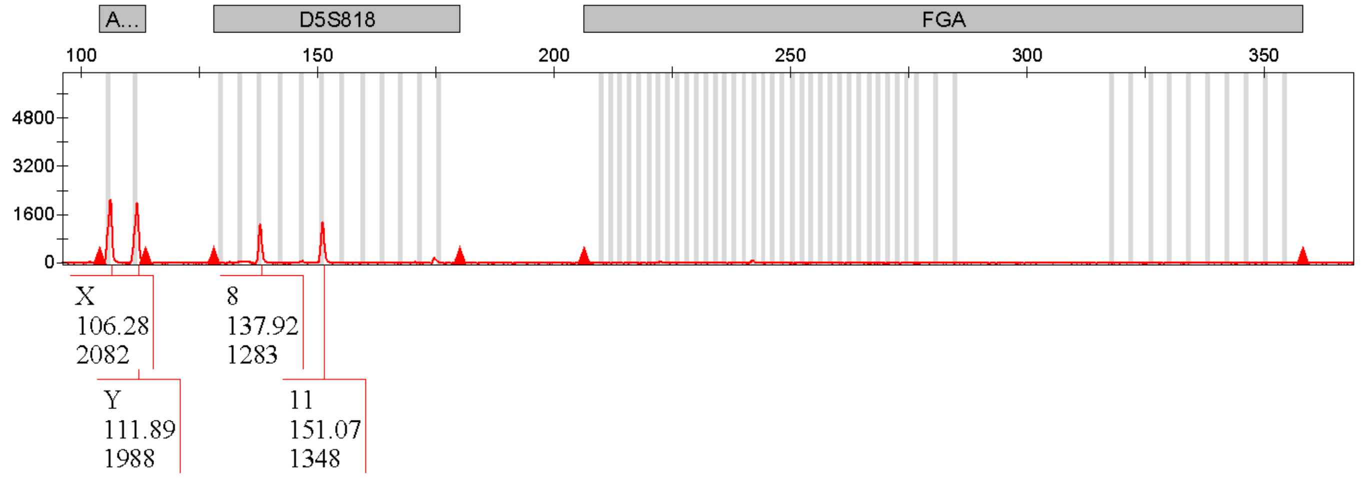

Supplement: Supplementary file 1 [file ijms-21-07888-s001.zip › ijms-972875 Figure S1.pdf]
