# Supplementary material for: Abnormal NFAT5 Physiology in Duchenne Muscular Dystrophy Fibroblasts as a Putative Explanation for the Permanent Fibrosis Formation in Duchenne Muscular Dystrophy
Source: Int J Mol Sci. 2020 Oct 24;21(21):7888. doi: 10.3390/ijms21217888 (PMC7660673; doi:10.3390/ijms21217888)
Supplement: Supplementary file 1 [file ijms-21-07888-s001.zip › ijms-972875-supplementary.pdf]

Supplementary Table 1. Cell culture data

Primary fibroblast culture	Status	Provided by	Age (years)	Gender	Mutation/ Deletion
UFibro	Healthy	Myobank (F)	17	Male	NA
DMDFibro	DMD	Myobank (F)	Unknown	Male	Exon 24 (mutation stop codon)

DMD= Duchenne muscular dystrophy.

Supplementary Table 2. Genes used in RT-qPCR

Gene	Primer	Concentration	Sour ce
NFAT5	PrimePCR SYBRGreen Assay qHsaCID0015734 intron-spanning	1x	Bio-Rad
UBC	F:ATTTGGGTCGCGGTTCTTG	1,25pmol	IDT
	R:TGCCTTGACATTCTCGATGGT	1,25pmol	IDT
HPRT1	F: TGACACTGGCAAAACAATGCA	1,25pmol	IDT
	R: GGTCCTTTTCACCAGCAAGCT	1,25pmol	IDT
B2M	F:TGCTGTCTCCATGTTTGATGTATCT	1,25pmol	IDT
	R: TCTCTGCTCCCCACCTCTAAGT	1,25pmol	IDT
RPL13A	F:CCTGGAGGAGAAGAGGAAAGAGA	1,25pmol	IDT
	R:CCTGGAGGAGAAGAGGAAAGAGA	1,25pmol	IDT
YWHAZ	F:ACTTTTGGTACATTGTGGCTTCAA	1,25pmol	IDT
	R: CCGCCAGGACAAACCAGTAT	1,25pmol	IDT
SDHA	F: TGGGAACAAGAGGGCATCTG	1,25pmol	IDT
	R: CCACCACTGCATCAAATTCATG	1,25pmol	IDT
HMBS	F: GGCAATGCGGCTGCAA	1,25pmol	IDT
	R: GGGTACCCACGCGAATCAC	1,25pmol	IDT
TBP	Unknown	1,25pmol	[27]
AluSq	Unknown	1,25pmol	[27]
AluSx1	Unknown	1,25pmol	[27]

UBC= ubiquitin C; *HPRT1*= hypoxanthine phosphoribosyltransferase 1; *B2M*= beta-2 microglobulin; *RPL13A*= 60S ribosomal protein L13a; *YWHAZ*= Tyrosine 3-Mono-oxygenase/Tryptophan 5-Mono-oxygenase Activation Protein, Zeta; *SDHA*= Succinate dehydrogenase complex, subunit A, *HMBS*= hydroxymethylbilane synthase, *TBP*= TATA-binding protein, *AluSq*= Alu restriction enzym, *AluSx1*= interspersed repeat subfamily. *NFAT5*= Nuclear Factor of Activated T-cells 5.

Supplementary Table 3. Primary antibodies used in Western-blotting

Antigen	Primary antibody	Clone	Concentration	Source
GAPDH	Mouse monoclonal IgM	71.1	0.4µg/mL	Sigma-Aldrich
NFAT5	Mouse monoclonalIgG2a	F-9	2µg/mL	Santa-Cruz Biotechnology

NFAT5= Nuclear Factor of Activated T-cells 5.

Supplementary Table 4. Primary antibodies used in immunocytohistochemistry

Antigen	Primary antibody	Clone	Concentration	Source
NFAT5 (V-18)	Goat polyclonal IgG	/	10 µg/mL	Santa-Cruz Biotechnology

NFAT5= Nuclear Factor of Activated T-cells 5; NCAM= neural cell adhesion molecule.
