# Supplementary figures and images for: Abnormal NFAT5 Physiology in Duchenne Muscular Dystrophy Fibroblasts as a Putative Explanation for the Permanent Fibrosis Formation in Duchenne Muscular Dystrophy
Source: Int J Mol Sci. 2020 Oct 24;21(21):7888. doi: 10.3390/ijms21217888 (PMC7660673; doi:10.3390/ijms21217888)

Supplementary Figure 2: Testing of NFAT5 Mouse specificity using siRNA

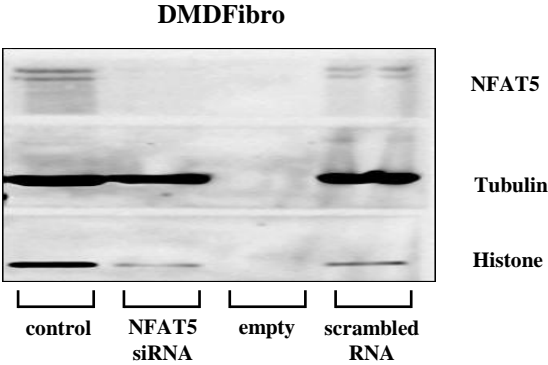

Supplement: Supplementary file 1 [file ijms-21-07888-s001.zip › ijms-972875 Figure S2.pdf]
